# Health Tract No. 311

**Published:** 1868-03

**Authors:** 


HEALTH TRACT No. 311

### DISCIPLINE OF LIFE.

The God of nature and of grace are one and the same embodiment of benevolence and wisdom and love, and "we his offspring are," his children and his heirs, hence the works and operations of nature and of grace never war against each other but act in harmony to the one great end, the elevation and happiness of the human family for time and for aye. If these first principles are kept constantly in view as a matter of firm religious faith and principle the sorrows of life, its disappointments and its tears would lose half their bitterness and the other half would be soon absorbed and forgotten in the contemplation of the great truth that "God is love."

We grow to mature age in the enjoyment of vigorous health; for half a life time we scarcely know what pain is; the physician has not been called to our dwelling; we have slept soundly, we have eaten heartily; we have been cozily housed in the bleak and weary winter time and for the summers in long succession we and ours have hied to the country, to the sea side and the spa, and have had our fill of enjoyment. But by this time we have begun to feel that we have a charmed life; that we owe all these grand blessings to a vigorous constitution and that that constitution is impregnable against accident, calamity and disease, and with such feelings and experiences "we have waxed fat and kicked" like Jeshuron of old; or like Nebuchadnezzar, we have given the glory of them all to our might, our wisdom, our habits of life. But one day we come home not quite so well as usual, and we feel tired and sad; the tea-table is as tidy as before; the children are as blithe and gladsome as usual; the fire burns brightly as ever, but all these fail to wake up the echos of loving joyousness as of old, and we retire to our beds with unspoken words; yet sleep comes not; but in its place restless tossings and the grumblings of approaching pains; anon the doctor is at our bedside; we look in his face, but there is no smile there; he makes a closer examination, but gives out no cheering word; he says nothing bad or good of the future, but unsolicited, promises to call in again in the course of an hour; this is ominous, and we begin to feel our strong foundation crumbling beneath us; the world, its pleasures, its appetites, its ambitions and its material interests fade away from our vision; and as the system becomes more oppressed by disease, more racked with pain, and with all, still sinking, sinking, sinking; then it is that we begin to wake up to an appreciation of past privileges and blessings, and sun-shine; of our dependence on the arm of the Infinite One, and our own helplessness and nothingness; and to that Power we stretch our withered arms for aid, and raise our feeble voice for succor, and he whose ear is ever open, and whose kind eye never sleeps, comes to our help, raises us up to health again and we are saved from wreck and ruin; and as often as we forget, other sicknesses come to wake us up again to a new sense of our dependence and helplessness and to the appreciation of the true source of all our blessings; and to the same end are we followed by moral disasters, by pecuniary reverses, by social sorrows, by domestic afflictions, all intended to lure us on by love, to brighter worlds on high; and thus it is that every pain and sob and sigh and tear is the "Discipline of Life" to wean us from the world and bind us more securely to the Everlasting One

